# Analysis of complications related to diabetic ketoacidosis in pediatric patients at a University Hospital: a cross-sectional study

**DOI:** 10.1016/j.jped.2025.01.009

**Published:** 2025-03-12

**Authors:** Caroline Quilice Naccarato, Nathalia Azevedo, Raphael Del Roio Liberatore

**Affiliations:** Universidade de São Paulo (USP), Faculdade de Medicina de Ribeirão Preto (FMRP), Departamento de Pediatria - Endocrinologia Pediátrica, Ribeirão Preto, São Paulo, Brazil

**Keywords:** Pediatrics, Diabetic ketoacidosis, Type 1 diabetes mellitus

## Abstract

**Objectives:**

Diabetic ketoacidosis is one of the main complications of type 1 diabetes mellitus and the leading cause of death among children and adolescents with the disease. The objective of this study was to characterize the cases of diabetic ketoacidosis treated in a University Hospital reference in pediatric endocrinology and identify their most frequent complications.

**Methods:**

A cross-sectional descriptive study was carried out, based on the review of medical records of patients aged 0–16 years with a diagnosis of diabetic ketoacidosis treated between January 2016 and August 2020. Insulin therapy was performed subcutaneously as part of the hospital's protocol.

**Results:**

Seventy-seven (77) admissions were analyzed and 55.8 % were diagnosed with a new case of type 1 diabetes. Adolescents (54.5 %) were the most affected. An increase of 90.9 % of cases between 2016 and 2020 was visualized. Severe DKA was more frequent in school-aged children. An increase in the dose of insulin was related to the severity of diabetic ketoacidosis. Hypokalemia was the most frequent complication. Cerebral edema occurred in 11.7 % of cases, and it was the cause of the only death, corresponding to a mortality rate of 1.3 %.

**Conclusions:**

Rising DKA incidence aligns with global trends, with poor adherence driving cases in previously diagnosed adolescents. High rates of hypokalemia and cerebral edema were found, but with lower mortality, showing the effectiveness of subcutaneous insulin for treatment. Future studies should confirm findings, address adherence issues, and refine hydration, insulin dosing, and monitoring practices to reduce complications.

## Introduction

Type 1 diabetes mellitus (T1DM) is one of the most prevalent chronic diseases in the pediatric population,^1^ characterized by autoimmune destruction of pancreatic beta cells, leading to total insulin deficiency.^2^ In 2021, Brazil was in third place in the world ranking of new cases of T1DM among individuals aged 0–19 years old.^1^ The incidence and prevalence have been increasing globally, due to rising disease cases and decreasing mortality.^1^

Diabetic ketoacidosis (DKA) is a serious and potentially life-threatening complication of T1DM and may be the first manifestation of the disease.^3^^,^^4^ It remains the main cause of death among children and adolescents with type 1 diabetes.^2^, ^3^, ^4^ The highest mortality is related to the occurrence of cerebral edema.^1^^,^^5^^,^^6^

The Clinical Hospital of the Ribeirão Preto Medical School (HC-FMRP), where the study was carried out, is a tertiary care center for pediatric endocrinology and pediatric emergencies in the XIII Regional Health Division of the state of São Paulo, comprising 26 cities. It is responsible for the treatment of DKA cases in the public health system. This study aimed to characterize the epidemiological and clinical features of pediatric DKA, evaluate its complications and morbidity, and evaluate the effectiveness of the treatment at the institution.

## Materials and methods

This is a cross-sectional descriptive study, based on the review of medical records from the Clinical Hospital of the Ribeirão Preto Medical School. The patient list was obtained from the Institution's Medical Archive Service, by searching for hospitalizations with diabetic ketoacidosis codes from ICD-10).^7^ Patients with multiple occurrences of DKA were included more than once.

The sample included pediatric patients (0 to 16 years old, which is the cutoff age for the pediatrics in this hospital) with DKA treated between January 2016 and August 2020. Charts from patients not meeting diagnostic criteria were excluded.

The project was approved by the Ethics and Research Committee of the HC-FMRP, by protocol number 5,832,639.

### Protocol for diagnosis and treatment of DKA in this service

The treatment of children and adolescents with DKA takes place in the Emergency Unit, a hospital designated for urgent and emergency cases. All children up to the age of 16 are treated by the pediatrics department, according to the institution's protocol. Patients older than this age are referred to the adult medical team. The pediatrics department follows its own DKA treatment protocol, which is unpublished and was developed by the leading emergency doctors of the sector.

In this protocol, DKA is defined as blood glucose above 200 mg/dl; venous serum pH below 7.3 and/or serum bicarbonate below 15 mmol/L; and presence of ketonemia and ketonuria.

DKA is classified as mild (pH between 7.3 and 7.2 or bicarbonate between 15 and 10 mEq/L), moderate (pH < 7.2 or bicarbonate between 5 and 10 mEq/L), and severe (pH < 7.1 or bicarbonate < 5 mEq/L).

Rehydration is made by intravenous 0.9 % saline solution. The degree of dehydration is clinically estimated between 6 and 8 % of body weight. Regular insulin administration starts after the first hour of hydration, using subcutaneous insulin therapy at a dose of 0.1 U/kg. The same dosage is administered every hour until the resolution of DKA (bicarbonate greater than or equal to 15 mEq/L and pH greater than or equal to 7.3). The diet is started after DKA is resolved.

Potassium chloride is added to the IV fluids when its serum level is below 6 mEq/L, with potassium infusion being 0.2 to 0.5 mEq/kg/h, according to the corrected potassium value. Insulin therapy is not indicated in patients with potassium levels of <3.0, requiring its replacement in higher doses (0.4 to 0.5 mEq/kg/h), and insulin started only after this correction.

The aim was to maintain blood glucose between 150 and 200 mg/dL, so when lower values, glucose was added to the IV fluids, or half the insulin dose (0.05 U/kg) could be given at certain times. Sodium bicarbonate is used only when pH values below 7 are found.

DKA cases were managed in the pediatric emergency room. Patients who need neurological monitoring (suspected or confirmed cerebral edema), ventilatory support, and difficult-to-control electrolyte disorders were admitted to Intensive Care Units.

### Evaluated data

In reviewing the medical records, the following aspects were analyzed: gender, age, type of diabetes mellitus (1 or 2), time since diagnosis (onset T1DM or previous diagnosis), treatment before admission to the HC-FMRP, initial tests (pH, bicarbonate (HCO3), blood glucose, sodium, potassium and phosphorus), time for recovery from DKA, the volume of infused solution and insulin used until recovery from acidosis, use of bicarbonate, the occurrence of complications (hypokalemia, hypoglycemia, hypophosphatemia, cerebral edema and death), and hospitalization in Intensive Care Units. The data were schematized in tables and figures, and it was calculated means, standard deviation, and frequencies using Microsoft Office Excel and the R program.

## Results

**Figure 1:**
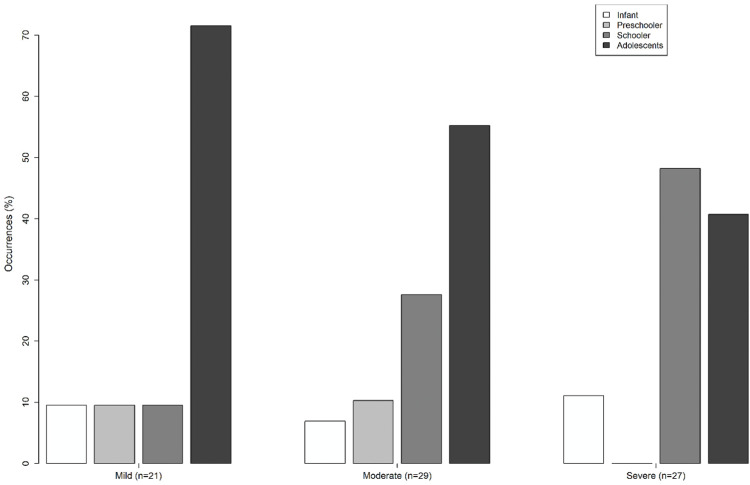
Frequency of mild, moderate, and severe cases by age range.

The final sample was 77 patients. Regarding the number of cases of DKA per year, the total was 11 in 2016, 13 in 2017, 12 in 2018, 20 in 2019, and 21 until August 2020, with an average of 15.4 cases per year, with an increase of 90.9 % between the first and last year of the study. In those patients, a similar distribution was observed between genders (50.6 % in males, 49.4 % in females). Among the evaluated cases, 55.8 % were diagnosed with a new case of T1DM. The most affected age group was adolescents (54.5 %). Detailed data can be seen in Table 1. Figure 1 shows the frequency of cases analyzed by age and DKA severity.

**Table 1:** General characteristics of the sample.

		Total	%
Gender	Male	39	50.6 %
	Female	38	49.4 %
Severity	Mild	21	27.3 %
	Moderate	29	37.7 %
	Severe	27	35 %
Diagnosis time	Onset	43	55.8 %
	Previous diagnosis	34	44.2 %
Age group	Infant (0–2 years old)	7	9 %
	Preschooler (2–4 years)	5	6.5 %
	School age (5–10 years old)	23	29.9 %
	Adolescents (11–16 years old)	42	54.5 %
Type of DM^a^	DM 1	75	97.4 %
	DM 2	2	2.6 %
Did they receive treatment prior to hospitalization?	Yes	42	54.6 %
	No	35	45.4 %

a Diabetes mellitus.

Regarding severity, it was classified as moderate in 37.7 % (*p* = 0,468) of the total, followed by severe cases in 35.1 % (*p* = 0,809) and mild in 27.3 % (*p* = 0.279). There was no statistically significant difference between the observed proportions for each severity level analyzed individually.

Comparing patients with DKA due to new-onset diabetes (43 cases) to those with a previous diagnosis (34 cases), the gender distribution was equal in both groups. The predominant age group in the new-onset diabetes cases was school-aged children (40 %), whereas adolescents accounted for the majority (79 %) in the previously diagnosed group. The severity of DKA was classified as severe in most cases in the new-onset group (44 %) and moderate in the decompensation group (41 %). Both groups exhibited high rates of complications, at 80 % and 70 %, respectively. In the new-onset group, one case (2 %) required ICU admission, compared to six cases (18 %) in the previously diagnosed group.

In the sample, seven patients (9.1 %) were admitted to the Intensive Care Units (UCI), with 1 being moderate (3.4 % of moderate DKA) and 6 severe (22.2 % of severe DKA). Within this group, 2 required an advanced airway. Regarding the moderate case, the patient's medical records did not provide justification for the ICU admission. It was a 15-year-old male patient who reported discontinuing insulin use as a suicide attempt. The entire management of the DKA was conducted in the ICU, despite the absence of cerebral edema, need for ventilatory support, or difficult-to-control electrolyte disturbances. Only one of the patients admitted to the ICU was a case of new-onset diabetes, while the others were already known to have the disease. Three patients were female (43 %) and four were male (57 %). Two patients were 9 and 10 years old, while the others were adolescents, aged 12 to 15 years.

The precipitating factors for DKA cases were an infectious condition in 39 % of the cases (with a predominance of respiratory infections in 69 %), poor adherence to treatment in previously diabetic patients in 21 % of the cases, and no acute cause identified in 40 %. Among patients who already had a diagnosis of diabetes, failure to use insulin was the cause in 47 % of cases of DKA.

The average time between the onset of symptoms and the diagnosis of DKA was 11.8 (± 16.4) days. The mean for DKA recovery time was 12.6 (± 16.1) hours, and the mean volume of infused solution was 78.7 (± 42.7) ml/kg. An increase in the insulin dosage and in the time to recovery was observed according to the greater severity of the cases, as shown in Table 2.

**Table 2:** Conduction of treatment.

	Mean recovery time (h^a^)	Mean volume infused (ml/kg)	Mean amount of insulin (IU/kg)
Mild	5.9	44.2	0.36
Moderate	8.7	70.4	0.61
Severe	21.9	116.2	1.07
Mean	12.6	78.7	0.70
SD^b^	16.1	42.7	0.51

a Hour.

b Standard deviation.

The use of bicarbonate was identified in 53.8 % of severe cases and 27.6 % of moderate cases, and it was not used for mild cases.

It was investigated the following complications: hypokalemia, hypoglycemia, cerebral edema, and death. The proportion of occurrence of each one of them, considering the total sample, and the proportion according to severity, can be seen in Table 3.

**Table 3:** Occurrence of complications according to the severity of the symptoms.

	Hypokalemia		Hypoglycemia		Cerebral edema		Death	
Severity of the DKA^a^:	Occurrences	%	Occurrences	%	Occurrences	%	Occurrences	%
Mild	7	33.3 %	4	19.1 %	0	0	0	0
Moderate	13	44.8 %	6	20.7 %	2	6.9 %	0	0
Severe	22	81.5 %	6	22.2 %	7	25.9 %	1	3.7 %
Total	42	54.5 %	16	20.8 %	9	11.7 %	1	1.3 %

a DKA, diabetic ketoacidosis.

Hypokalemia was the most frequent complication during treatment, occurring in 54.5 % of cases, and occurring in 81.5 % of severe DKA. Hypoglycemia (values below 70 mg/dL) occurred in 20.8 % of cases, with a similar distribution according to severity.

Regarding hypophosphatemia, inorganic phosphorus levels were found in only 33.8 % of admissions. Within this percentage, there were 21 cases of hypophosphatemia (80.8 %).

The occurrence of cerebral edema (all confirmed by head computed tomography) was found in 11.7 % of the total (9 patients), being more frequent in severe cases. It was found that 40.2 % of children had neurological symptoms during DKA correction, and of these, 29 % received confirmation of edema by imaging. The symptoms of cerebral edema presented were drowsiness (100 % of cases), alteration in the Glasgow Coma Scale (88.9 % of cases), headache (44.5 %), hyporesponsiveness (44.5 %), dizziness (11 %), and agitation (11 %).

Cerebral edema was the cause of the only death, corresponding to an overall DKA mortality of 1.3 %. Considering mortality only among cases of edema, this corresponds to 11.1 %.

## Discussion

Over the study period, the authors observed a significant increase in DKA cases, with nearly double the number of cases reported in 2020 compared to 2016, especially considering that the cases analyzed in 2020 are only up to August. This trend aligns with global studies indicating rising DKA incidence, such as reports from Germany, China, Chile, and Croatia.^8^, ^9^, ^10^, ^11^ Potential factors contributing to this increase include delayed diagnosis, limited public awareness of T1DM symptoms, and healthcare access challenges exacerbated by systemic inequalities.^12^

Among the cohort, 55.8 % of DKA cases occurred in newly diagnosed T1DM patients, consistent with other Brazilian studies reporting rates between 41 % and 59.6 %.^13^, ^14^, ^15^ Data from the world literature show a wider variation: it ranges from 32 % to 67,4 %.^9^^,^^11^^,^^16^, ^17^, ^18^ Such variability is related to the incidence of type 1 diabetes in each region and public health initiatives for population education for early recognition of disease symptoms.^10^

Regarding the age group (Table 1), the authors identified most cases in adolescents (11–16 years, 54.5 %), mostly with mild symptoms, like studies in Paraguay (57.9 % in adolescents).^6^ A greater number of cases of DKA was also found in adolescents who already had a diagnosis of type 1 diabetes in a Chilean study, associated with poor adherence to treatment^11^; in Venezuela, cases in previously diabetic patients were also higher in the 10–19 age group.^16^ This contrasts with severe DKA being more prevalent among school-aged children (Figure 1), likely due to delays in diagnosis related to nonspecific symptoms.

In this series, 64 % of the cases of adolescents already had a diagnosis of type 1 diabetes. From this number, 49 % presented a lack of adequate use of insulin as the cause of DKA, even with clinical follow-up in specific outpatient clinics and free medication for treatment. Among the other age groups with a previous diagnosis of diabetes, only 16 % of DKA were due to poor adherence to therapy.

The present findings indicate that recovery times for DKA in the studied institution (mean 12.6 h) exceed those reported by the International Society for Pediatric and Adolescent Diabetes (ISPAD) (mean 11.6 h),^19^ especially in more severe cases (Table 2). In other Brazilian series, the authors found an average for recovery ranging from 11 to 27 h,^14^^,^^15^ with a difference in the time for DKA resolution considering the normalization of pH and bicarbonate individually, with an average of 18 h for the first and 21 h for the second.^15^ In other countries, it takes about 14 to 29 h to correct DKA.^10^^,^^20^^,^^21^

This discrepancy may reflect differences in treatment criteria. The protocol requires both pH ≥ 7.3 and bicarbonate ≥ 15 mEq/L to define resolution, while others take into account only one or the other.^3^^,^^10^^,^^22^

Prolonged treatment time is directly associated with the use of larger volumes of hydration and insulin, which can lead to the appearance of complications such as hypoglycemia and hypokalemia. In comparison with a study in Campinas (São Paulo, Brazil) in which the total volume used for the treatment of severe cases was on average 82.7 ml/kg in moderate cases and 71.4 ml/kg in mild cases,^15^ in this service the total volume needed was lower in mild and moderate cases (44.1 and 70.4 respectively), and higher in severe cases (116.2 ml/kg).

Regarding the use of insulin, there was a greater need for insulin therapy, the greater the severity of the case. This finding is similar to the study carried out in Campinas, in which the time of insulin administration was directly proportional to the severity of the cases.^15^ However, the institutional protocol makes use of subcutaneous regular insulin every hour for the treatment of DKA, unlike other evaluated studies.^14^^,^^15^^,^^23^

As for the use of sodium bicarbonate, its use is not routinely recommended.^3^^,^^18^^,^^23^^,^^24^ Studies have not shown benefit from the administration of bicarbonate, with its use being suggested only in cases of severe acidosis (pH < 6.9) associated with cardiac alteration or life-threatening hyperkalemia.^3^ Its use was carried out in 0.04 % of hospitalizations in Campinas, all of which were severe cases of patients in the Intensive Care Units who did not respond to the initial treatment.^15^ In the study carried out in Paraguay, it was used in 5 % of the cases.^20^ In this work, higher use was identified in moderate and severe cases refractory to initial treatment.

Regarding complications, hypokalemia was the most frequent complication (Table 3). In other study from Brazil, 34.6 % of the patients had hypokalemia.^14^ In worldwide data, the frequency of hypokalemia ranged from 22 % to 67,1 %.^11^^,^^25^, ^26^, ^27^, ^28^ Research suggests that the use of lower doses of insulin leads to a lower risk of developing hypokalemia.^28^

Hypoglycemia was the second most common complication found in this work, occurring in 20.8 % of cases. In other Brazilian studies, the rate was lower, ranging from 13.1 % to 15 %.^14^^,^^15^ The authors must consider that the difference in the frequency of hypoglycemia between the services may be due to the use of different cuts for its definition; in this protocol, it is defined as values below 70 mg/dL. Studies show the occurrence of hypoglycemia in about 25 % of DKA treatments using high doses of insulin and indicate that this frequency could decrease with continuous insulin infusion at lower doses.^26^

Hypophosphatemia is a disorder little researched in the service, and that may be underdiagnosed. However, there is no clinical proof of benefit in the treatment with its replacement.^3^

In the present data, the authors found an ICU admission rate of 9.1 % among all cases and an admission rate of 22 % when considering only severe cases. ISPAD recommends ICU admission for all severe DKA cases or those at high risk for cerebral edema. However, there is limited published data on ICU admission rates for children during DKA treatment. A recent publication by an Italian group reported a total ICU admission rate of 10 % in severe DKA cases.^29^ Meanwhile, another recent monocentric study from Croatia showed bigger rates.^10^ Discrepancies in DKA-related hospitalization rates may be influenced by unequal access to PICU resources across regions or by differences in the proficiency of DKA management in non-intensive pediatric care units.

The occurrence of cerebral edema reported by ISPAD ranges from 0.5 to 0.9 %.^3^ In this study, the authors found a rate of 11.7 %, which is considered high. In other recent publications in Brazil, the frequency of cerebral edema ranged from 1.24 % to 5.7 %.^13^, ^14^, ^15^ In other countries, it ranged between 1.2 % and 13.2 %.^10^^,^^21^^,^^25^

According to ISPAD, the number of deaths due to DKA in children ranges from < 1 % in developed countries to 3–13 % in developing countries, and from 21 % to 24 % among those with cerebral edema.^3^ In this research, among all patients, the authors found a death rate of 1.3 %, which was higher than reported by current guidelines. Regarding mortality among cases with cerebral edema, the rate found was 11 %, lower than that reported by ISPAD. In other Brazilian studies, it ranged from 0.62 % to 2 % of the total of patients.^13^, ^14^, ^15^ In Campinas, it corresponded to 100 % among cases with cerebral edema.^15^ In other countries, the frequency of deaths ranged between 0.6 % and 13.2 % of the total patients, and, from this total, 15 %–33.5 % occurred due to cerebral edema.^10^^,^^21^^,^^26^

The objective of this study was to characterize the pediatric population with DKA at HC-FMRP and its complications. Increasing DKA incidence rates were observed, consistent with other studies in Brazil and worldwide. Adolescents, many with previously diagnosed diabetes, were the most affected, highlighting adherence difficulties. Cerebral edema rates were higher than expected, but mortality was lower, emphasizing the need for preventive measures.

Further studies are necessary to explore the 2020 DKA increase and confirm its continuity. Complication data from this study can guide improvements in protocols, focusing on reducing hypoglycemia and hypokalemia. Notably, the low mortality rate among cerebral edema cases suggests subcutaneous insulin is a viable alternative where intravenous pumps are unavailable.

The study's limitations include retrospective analysis of non-standardized medical records, which complicates data collection. Additionally, patients often receive external treatment that lacks detailed records, affecting the assessment of pre-hospitalization therapeutic measures. While the study's sample size of 77 patients may be low, the HC-FMRP serves as a regional reference for pediatric endocrinology, enabling the collection of numerous cases and allowing for protocol evaluation with other global guidelines. Despite these challenges, the study's findings provide critical insights into local DKA management and allow actionable improvements in treatment protocols, including optimizing hydration strategies, insulin dosing, and complication monitoring.

## Funding

This research did not receive any specific grant from funding agencies in the public, commercial, or not-for-profit sectors.

## Conflicts of interest

The authors declare no conflicts of interest.
